# Uterine cervical Müllerian adenosarcoma possibly arising from ovarian cystadenofibroma: A case report and review of the literature

**DOI:** 10.3389/fonc.2022.1064851

**Published:** 2023-01-04

**Authors:** Xinxin Zhu, Chao Peng, Yan Huang, Yingfang Zhou

**Affiliations:** ^1^ Department of Obstetrics and Gynecology, Peking University First Hospital, Beijing, China; ^2^ Department of Obstetrics and Gynecology, Peking University First Hospital Ningxia Women and Children's Hospital (Ningxia Hui Autonomous Region Maternal and Child Health Hospital), Yinchuan, China

**Keywords:** Müllerian adenosarcoma, cystadenofibroma, clinicopathological characteristics, differential diagnosis, prognosis

## Abstract

Uterine cervical Müllerian adenosarcoma (MA), a rare malignant tumor of the female reproductive organs, is composed of a benign epithelium and a low-grade malignant stromal component. Because few studies have investigated the clinical management of MA, misdiagnosis often occur. Therefore, we proposed an optimal course of clinical management for patients with MA. MA is possibly a malignant transformation of the cystadenofibroma. In this study, we present a case of a 46-year-old woman who presented with symptoms of MA of the uterine cervix, such as metrorrhagia and a cyst in the cervical canals, after transvaginal excision of the left ovarian mucinous cystadenofibroma.

## 1 Introduction

Müllerian adenosarcoma (MA) is a malignant tumor composed of benign epithelial glands and a malignant sarcomatous stromal component. MA was first identified by Clement and Scully in 1974 (1). MA is a rare tumor accounting for approximately 2% of all instances of adenosarcomas of the female reproductive organs. In addition, MA can be found in different locations of the uterine corpus (2). Contrary to most cervical malignant tumors, no association with the human papillomavirus (HPV) has been reported (3). MA occurs in postmenopausal and young women; however, the average age of patients with MA is lower than that of patients with uterine corpus tumors. The symptoms of cervical MA include vaginal bleeding or a cervical mass/polyp, which can be detected during routine gynecological examination (4). However, the origin of cervical MA is unclear. Coverage of these cases is scant in the case report literature, and few studies have investigated the occurrence, diagnosis, and clinical management of MA; thus, misdiagnosis often occur. In this study, we report a case of a 46-year-old premenopausal woman with cervical MA after transvaginal excision of the left ovarian mucinous cystadenofibroma. A previous study proposed that MA is a malignant transformation of the cystadenofibroma (5). We corroborated the finding of the previous study and speculated that the transvaginal excision of the ovarian tumor may lead to cervical MA. According to our review of the literature, this is the first reported case of cervical adenosarcoma arising from a benign ovarian cystadenofibroma.

### 1.1 Case

We treated a 46-year-old married woman who presented with a cervical cyst that had been growing for 2 years with irregular vaginal bleeding for the past 6 months in our hospital. She underwent a transvaginal excision of the left ovarian cyst in 2005. The postoperative pathology results of the left ovarian tumor showed a mucinous cystadenofibroma. Subsequently, the patient underwent a laparoscopic excision of the left adnexal pseudocyst about 10 cm and pelvic adhesion lysis in 2007. The postoperative pathology results indicated left adnexa in the fibrous connective tissue.

**Figure 1:**
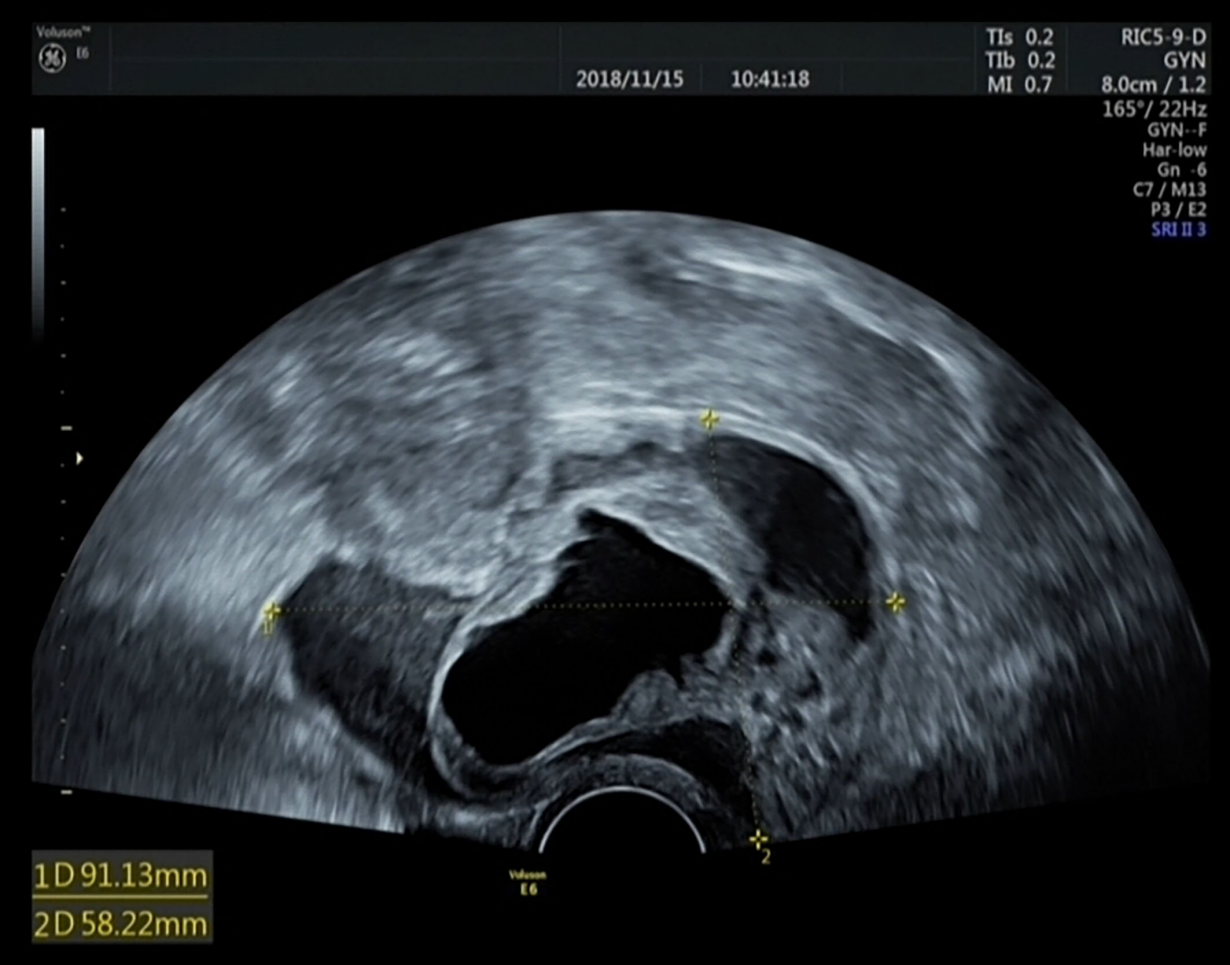
Ultrasound results of the tumor. Müllerian adenosarcoma of the uterine cervix is a cystic-solid mass measuring 9.1 × 8.1 × 5.8 cm.

The examination results showed that the diameter of the mass in the cervix was 4 cm and an enlarged uterus, the size of 8-week’s gestation (8.9 × 4.4 × 3.4 cm). Ultrasonography revealed a cystic-solid mass in the cervix measuring 9.1 × 8.1 × 5.8 cm (**Figure 1**) and a solid-cystic mass measuring 3.6 × 2.9 × 2.7 cm in the posterior uterine wall. The test for HPV 66 was positive (6), and a biopsy of the colposcopy revealed cervicitis. The computed tomography (CT) scan showed that tumor did not extend posteriorly to the bladder or rectum and the pelvic para-aortic lymph nodes were not enlarged. Abdominal/pelvic ultrasound and chest radiography indicated no abnormalities. Total laparoscopic hysterectomy and right salpingo-oophorectomy were performed on the basis of the patient’s age, ultrasound results, HPV status, and past surgery history. We chose a watch-and-wait strategy to observe the patient. The patient is currently disease-free after a 48-month follow-up period.

### 1.2 Pathology findings

**Figure 2:**
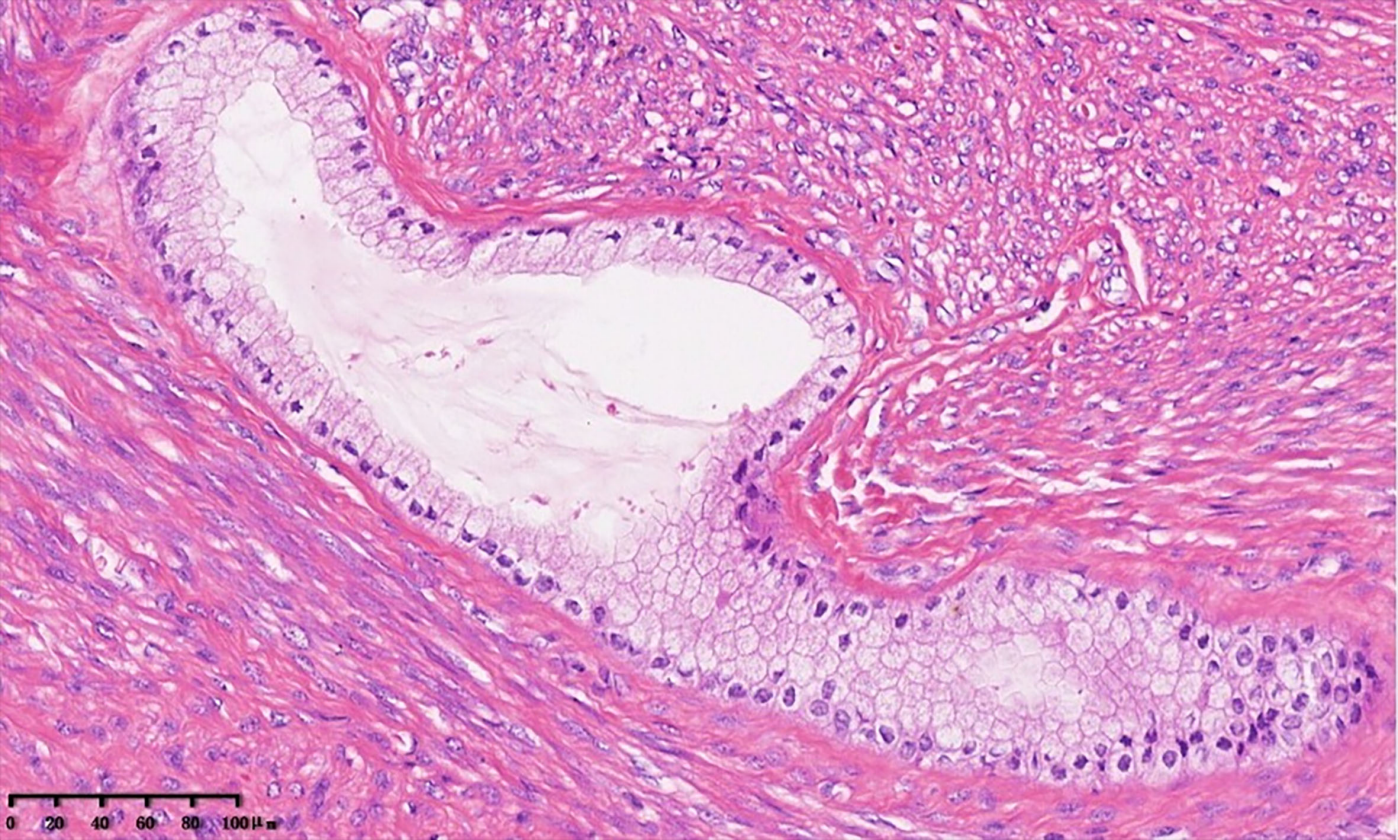
Müllerian adenosarcoma of the uterine cervix. The tumor shows benign columnar glands and low-grade endometrial stromal sarcoma of the cervix. The glands were surrounded by a cellular stroma that formed periglandular cuffs (×20).

**Figure 3:**
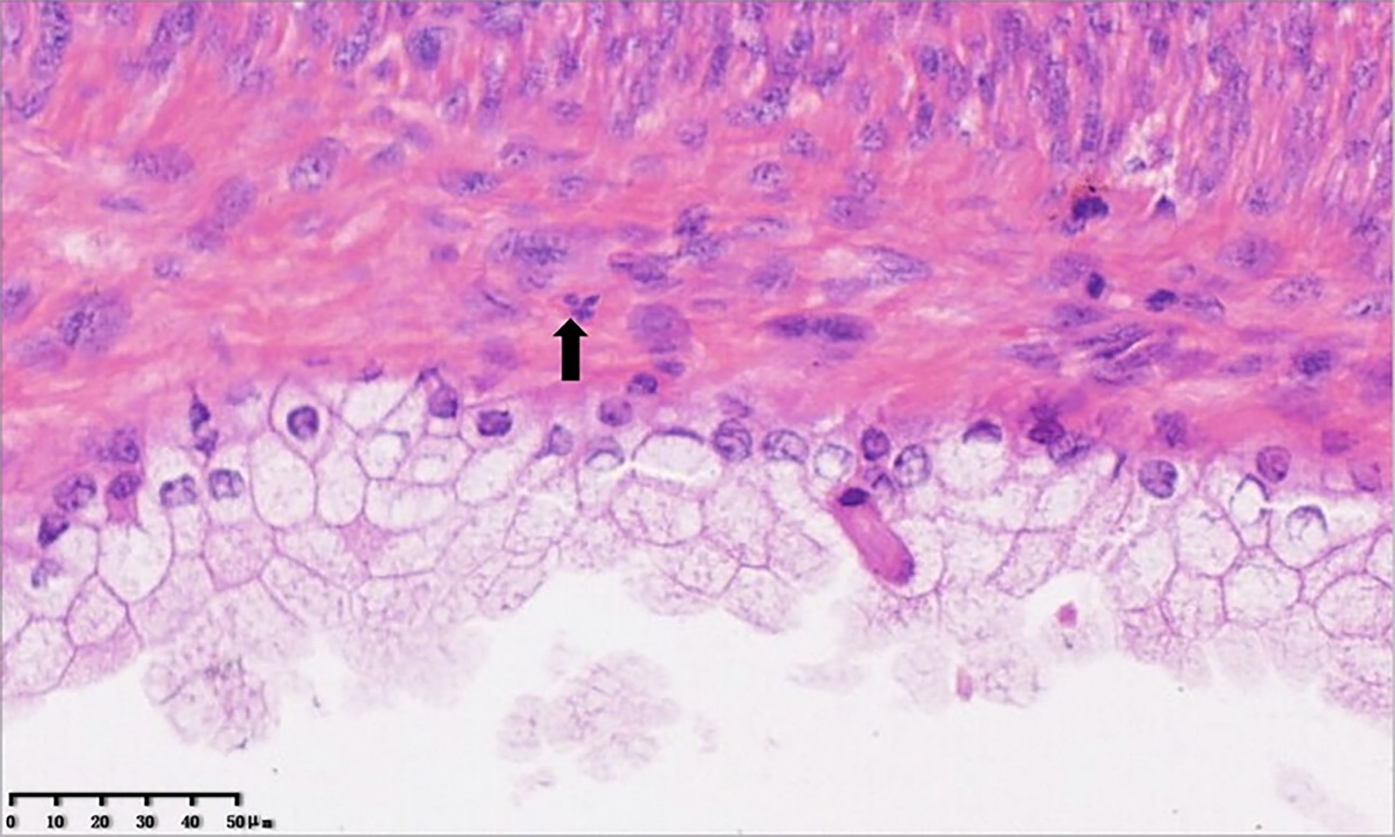
Cellular stroma composes of atypical mitotically active spindle cells (×40).

The specimen of total laparoscopic hysterectomy with right salpingo-oophorectomy was received for histopathological examination. On gross pathological examination, a multilocular cystic mass measuring 5.0 × 4.8 × 4.5 cm was observed in the cervix. The cut surface of the tumor was a mucosal surface. A 1.5-cm myoma was observed in the myometrium of the posterior uterine wall. The endometrial cavity was free of tumors. The right ovary and the right fallopian tube were normal. On microscopic examination, the mass was found to be benign or mildly atypical Müllerian glands and low-grade malignant stroma (**Figure 2**). Benign and atypical endocervical glands were uniformly distributed within the tumor, and most of them had a cystic appearance. The glands were covered with a cellular stroma that formed periglandular cuffs and intraluminal polyploid projections. The stromal component focally resembled a low-grade stromal sarcoma covering 20% of the tumor area. In the low-grade sarcomatous areas, the mitotic rate was low (3 mitotic figures/10 high power fields (HPFs) (**Figure 3**) (7). An immunohistochemical staining analysis of various markers revealed that P16 was partially positive; Ki67 (15%), ER, PR, MUC1, CK7, CA125, and Pax8 were positive; and P53, CDX2, MUC5, CK20, and CEA were negative. Myoma was observed in the myometrium of the posterior uterine wall; however, the right ovary and the right fallopian tube were free of tumors. On the basis of these histopathological features, MA was diagnosed in accordance with the International Federation of Gynecology and Obstetrics criteria (8).

## 2 Discussion

MA is a mixed epithelial and mesenchymal tumor with benign or mildly atypical glandular and malignant stromal cells. In total, 71%, 15%, 12%, and 2% of cases had MA occurring in the uterine corpus, ovaries, pelvis, and uterine cervix, respectively (9). The etiology of MA remains unknown because of its rarity. According to previous studies, MA has not been associated with HPV infection. However, in our study, the patients tested positive for HPV 66. Patients with MA vary widely in age but are often younger than patients with uterine corpus tumors. A report on primary cervical adenosarcomas was published by Jones and Lefkowitz (10). In their study covering 12 cases and a review of 12 articles, the average age of patients was 31 years (11–65 years), with one-third under the age of 15 years, and the tumor size varied from 1.0 to 4.5 cm. In most of the cases reviewed, patients presented with abnormal vaginal bleeding, abdominal or pelvic pain, and a cervical mass/polyp. In our study, the woman was of the reproductive age and presented with vaginal bleeding as her major symptom.

Mixed Müllerian tumors are classified into adenomyomas, adenofibromas, adenosarcomas, and carcinosarcomas (malignant Müllerian mixed tumors) on the basis of the composition of benign or malignant epithelial and mesenchymal elements of Müllerian origin. MA is a low-grade neoplasm along the spectrum of mixed Müllerian tumors, with adenofibromas at the beginning and carcinosarcomas at the end (11). Some tumors currently classified as adenofibromas on the basis of their low mitotic count and lack of nuclear atypia are well-differentiated adenosarcomas. Indeed, adenofibroma from adenosarcoma are difficult to distinguish, and the two tumors have a somewhat similar appearance at low magnification. As a result, a confirmed diagnosis of adenofibroma cannot be made on curetted material, and a hysterectomy is required to ensure adequate sampling and exclude adenosarcoma. The most helpful criterion for distinguishing adenosarcoma from adenofibroma is the frequency of mitotic figures found in the stroma. Mitotic activity greater than 1 per 10 HPFs warrants a diagnosis of adenosarcoma (8). The practical approach is to classify as adenosarcoma any biphasic tumor with atypical hypercellular stroma, periglandular stromal cuffing, and one or more mitosis per 10 HPFs (7). Immunohistochemistry may have no utility in differentiating adenosarcoma from benign masses. In addition, previous studies have reported that a patient had an extrauterine pelvic Müllerian adenosarcoma that recurred on multiple occasions and was originally diagnosed as a benign lesion (12, 13). Thus, caution is needed in the initial classification of such benign lesions as adenofibromas. In this study, the patient underwent pelvic surgeries, one in 2005 and another one in 2007. In 2005, she underwent a transvaginal excision of the left ovarian cyst, which revealed a mucinous cystadenofibroma. A previous study proposed that MA is a malignant transformation of the cystadenofibroma (5). In our study, we propose that adenofibromas serve as precursors of Müllerian adenosarcoma in some cases and that transvaginal surgery may promote the occurrence of cervical MA. Transvaginal surgery is a minimally invasive surgery and is currently the preferred treatment for symptomatic ovarian cysts because of its three advantages: less postoperative pain, fewer postoperative complications (i.e., incisional hernia), and improved cosmetic satisfaction. However, despite the popularity of minimally invasive surgery, preoperative evaluation of the ovarian mass is necessary (14).

The management of adenofibroma and adenosarcoma is assumed to be similar and includes a hysterectomy. However, the optimal course of therapy for adenosarcomas is also uncertain. This uncertainty may arise from the fact that adenosarcoma appears in the early stages of reproductive life, and its potential for malignancy remains poorly understood. Most authors have recommended hysterectomy accompanied by bilateral salpingo-oophorectomy because treatment plans are made by analogy with treatments for common diseases for which the optimal treatment has already been determined (15). Although salpingo-oophorectomy is generally recommended, the strength of the evidence for or against ovarian conservation remains insufficient (16). In our study, because the patient had previously undergone laparoscopic left adnexectomy, a total laparoscopic hysterectomy with right salpingo-oophorectomy was performed. Local excision has been curative in rare cases and can be performed in young patients with pedunculated cervical tumors and uninvolved stalks, which allows the conservation of reproductive function (17). However, postoperative recurrences after 5 years are not unusual (18). In a series of 100 cases, Clement and Scully (7) reported that the recurrence rate of MA after surgery was 23.9% and that one-third of the recurrences occurred after 5 years. Therefore, a long-term follow-up period of more than 5 years is necessary for adequate surveillance.

The prognosis of MA is characterized by invasion of the cervical wall and sarcomatous overgrowth (SO). Although MA is regarded as less aggressive and has lower malignancy potential, some unfavorable prognostic factors have been reported, such as a high mitotic rate and the presence of heterologous elements, deep myometrial invasion, necrosis, and extrauterine spread. However, the presence of myometrial invasion and SO has been demonstrated to be associated with poor prognosis, increased risk of postoperative recurrence, and fatal outcome (7, 19). Fortunately, in our study, the patient was diagnosed with MA.

We conducted a Medline search for articles on MA published in English between 1976 and 2022 using “Müllerian adenosarcoma, uterine cervix” or “adenosarcoma, uterine cervix” or “Müllerian adenosarcoma, cervix” or “adenosarcoma, cervix” as keywords and selected papers reporting data on premenopausal women. The clinical characteristics of these patients are summarized in **Table 1**. We can conclude from the table that cervical MAs are rare tumors and tend to appear more often in younger women. Most patients presented with vaginal or cervical masses and vaginal bleeding. Hysterectomy with salpingo-oophorectomy or fertility-sparing surgery was performed depending on the patient’s age, bearing requirement, and stage of the disease. Kanayama et al. (36) reported that normal fertility is not affected after conservative surgery. Myometrial invasion and SO are major prognostic factors. Therapies should be planned based on a patient’s condition. Recurrences may occur late; therefore, long-term follow-up is necessary. To our knowledge, this is the first reported case of cervical adenosarcoma arising from a benign ovarian cystadenofibroma, furthermore, more studies are recommended to clarify it.

**Table 1:** Clinicopathologic characteristics of Müllerian adenosarcoma of the cervix in chronological order.

Reference	Age	Parity	Presenting symptoms	Surgery	SO	Adjuvant therapy	Status(months)
Roth et al. (1976) (20)	14	0	Watery discharge	RH + partial vaginectomy+BSO+PLD	NM	Radiotherapy +chemotherapy	NED (42)
Ostor and Fortune (1980) (21)	19 47	NM NM	Vaginal discharge Metrorrhagia	Polypectomy TAH+BSO	No No	No No	AWD (132) NED (5)
Zaloudek et al. (1981) (18)	15	NM	Mass	Local excision	No	No	NED (48)
Gal et al. (1988) (22)	14	0	Mass	TAH+BSO	Yes	Chemotherapy+radiotherapy	DOD (18)
Gast et al. (1989) (15)	15	0	Mass	RH	No	No	NED (48)
Jones et al. (1995) (10)	30	NM	Mass	TAH + BSO	No	Chemotherapy	NED (27)
Ramos et al. (2002) (23)	25	0	Metrorrhagia	TAH+BSO	NM	No	NED (24)
Park et al. (2004) (24)	37	NM	Vaginal spotting	TAH+BSO+PLD	Yes	No	NED (9)
Manoharan et al. (2007) (2)	26 28 43	2 0 1	Intermenstrual and postcoital bleeding Postcoital bleeding Postcoital bleeding	Vaginal hysterectomy + PAND RH TAH	No Yes No	Radiotherapy Chemotherapy+radiotherapy No	NED (36) NED (48) NED (NM)
Fleming et al. (2009) (25)	10	0	Vaginal bleeding	RH+BS+PLD+upper vaginectomy	NM	No	NED (NM)
Bagga et al. (2010) (26)	15	0	Vaginal bleeding	TAH+BSO+PLB+partial omentectomy	No	No	NED (9)
Buyukkurt et al. (2010) (27)	14	0	Mass	Excisional biopsy	NM	No	NED (15)
Duggal et al. (2010) (28)	15	0	Foul smelling and menstrual bleeding	TAH+BSO+ omentectomy	Yes	Chemotherapy+radiotherapy	DOD (12)
Charfi et al. (2012) (29)	26	0	Mass	TAH	Yes	No	Unknown
Chin et al. (2013) (30)	17	0	Vaginal bleeding and introital mass	Cervical wedge resection	No	No	NED (204)
Sanamandra et al. (2014) (31)	13	0	Vaginal mass and vaginal bleeding	Tumor resection	NM	No	Unknown
Seagle et al. (2014) (32)	54 47	NM NM	Vaginal bleeding Vaginal bleeding	RH+BSO+PLD+PAND Cervical biopsies	Yes Yes	Chemotherapy+radiotherapy Chemotherapy+radiotherapy	NED (66) DOD (12)
Podduturi et al. (2016) (33)	38	3	Postcoital bleeding	NAC+ RH + BSO + PLD	Yes	No	NED (10)
Morales et al. (2016) (34)	39	NM	Pelvic pain + vaginal bleeding	RH+PLD	Yes	No (patient rejection)	AWD (21)
Shinnick et al. (2017) (35)	14	0	Mass	CKC	NM	No	Unknown
Kanayama et al. (2017) (36)	28	0	Mass	CKC	No	No	NED (32)
Koyuncuoğlu et al. (2017) (37)	32	2	Vaginal bleeding	TAH+BSO	Yes	Chemotherapy+radiotherapy	Unknown
Togami et al. (2018) (38)	32 34 17	0 0 0	Vaginal bleeding Vaginal bleeding Vaginal bleeding and introital mass	TAH+BS MRH+BSO+PLD+LAR Conization	No No No	No Pazopanib No	NED (28) NED (13) NED (62)
Yuan et al. (2019) (39)	19 32	0 0	NM NM	Hysteroscopy+ TR Hysteroscopy+ TR	Yes Yes	Chemotherapy Chemotherapy	NED (13) NED (10)

AWD, alive with disease; BS, bilateral salpingectomy; BSO, bilateral salpingo-oophorectomy; CKC, cold knife conization; DOD, dead of disease; LAR, low anterior resection; MRH, modified radical hysterectomy; NAC, neoadjuvant chemotherapy; NED, no evidence of disease; NM, not mentioned; PAND, para-aortic node dissection; PLB, pelvic lymph node biopsy; PLD, pelvic lymph node dissection; RH, radical hysterectomy; TAH, total abdominal hysterectomy; TR, tumor resection.

## Data availability statement

The raw data supporting the conclusions of this article will be made available by the authors, without undue reservation.

## Ethics statement

The studies involving human participants were reviewed and approved by Peking University First Hospital’s ethical review committee. The ethics committee waived the requirement of written informed consent for participation.

## Author contributions

XZ: conceptualization and writing-original draft. CP: data analysis. YH: writing-original draft. YZ: conceptualization, writing-reviewing and editing. All authors contributed to the article and approved the submitted version.
